# Antifouling Compounds from Marine Macroalgae

**DOI:** 10.3390/md15090265

**Published:** 2017-08-28

**Authors:** Hans Uwe Dahms, Sergey Dobretsov

**Affiliations:** 1 Department of Biomedical Science and Environmental Biology, Kaohsiung Medical University, No. 100, Shin-Chuan 1st Road, Kaohsiung 80708, Taiwan; hansd@kmu.edu.tw; Tel.: +886-(0)7-312-1101-2695; 2 Department of Marine Biotechnology and Resources, National Sun Yat-sen University, No. 70, Lienhai Road, Kaohsiung 80424, Taiwan; 3 Research Center for Environmental Medicine, Kaohsiung Medical University, Kaohsiung 80708, Taiwan; 4 Center of Excellence in Marine Biotechnology, Sultan Qaboos University, Muscat 123, Oman; 5 Department of Marine Science and Fisheries, College of Agricultural and Marine Sciences, Sultan Qaboos University, Muscat 123, Oman

**Keywords:** macroalgae, antifouling, biofouling, biogenic compounds, quorum sensing, marine natural products

## Abstract

Marine macroalgae produce a wide variety of biologically-active metabolites that have been developed into commercial products, such as antibiotics, immunosuppressive, anti-inflammatory, cytotoxic agents, and cosmetic products. Many marine algae remain clean over longer periods of time, suggesting their strong antifouling potential. Isolation of biogenic compounds and the determination of their structure could provide leads for the development of environmentally-friendly antifouling paints. Isolated substances with potent antifouling activity belong to fatty acids, lipopeptides, amides, alkaloids, lactones, steroids, terpenoids, and pyrroles. It is unclear as yet to what extent symbiotic microorganisms are involved in the synthesis of these compounds. Algal secondary metabolites have the potential to be produced commercially using genetic and metabolic engineering techniques. This review provides an overview of publications from 2010 to February 2017 about antifouling activity of green, brown, and red algae. Some researchers were focusing on antifouling compounds of brown macroalgae, while metabolites of green algae received less attention. Several studies tested antifouling activity against bacteria, microalgae and invertebrates, but in only a few studies was the quorum sensing inhibitory activity of marine macroalgae tested. Rarely, antifouling compounds from macroalgae were isolated and tested in an ecologically-relevant way.

## 1. Introduction

Biofouling is the undesirable growth of micro- (bacteria and protists) and macro-fouling (invertebrates and algae) organisms on submerged surfaces [1]. Clean substrates in the marine environment will be quickly fouled by such organisms [2,3]. Biofouling of marine installations costs billions of US dollars for the maritime industry and navies around the world [4]. The majority of antifouling technologies use toxic biocides, like copper, that kill organisms and accumulate in the environment [4,5,6]. While there are non-toxic antifouling methods on the market, they are costly and not as effective as traditional biocidal solutions [4,5]. Thus, new non-toxic antifouling methods are urgently needed.

The term biofouling is applicable not only to man-made structures but to marine organisms. Several studies state that most marine organisms stay relatively free from biofouling [3]. This suggests that these organisms have evolved different antifouling strategies. By investigating such strategies, we may find and develop new antifouling methods.

How can marine organisms defend themselves from biofouling? Surface wettability and surface microstructures were proposed as critical antifouling mechanisms [3,7]. Many organisms excrete secondary metabolites that repel or deter biofouling species [8]. Additionally, it was shown that specific microorganisms associated with surfaces of marine algae, sponges, and corals can prevent the colonization of hosts by other fouling organisms [2,3,5]. In most cases, marine species rely not only on one mechanism, but use combined physical, chemical, and biological strategies to prevent biofouling [3].

Macroalgae represent a large multicellular polyphyletic group of photosynthetic eukaryotic organisms [9]. Classification of seaweed follows the genealogy of their plastids. The main groups include green algae (division Chlorophyta), brown algae (division Phaeophyta) and red algae (division Rhodophyta) [9]. Some investigators also include prokaryotic blue-green algae (phylum Cyanobacteria), which are excluded from this review. Marine macroalgae are quite dominant in polar, temporal, and tropical waters [9]. Large biomasses of drifting marine macroalgae are wasted and only a few species are currently in use as human foods, cosmetics, fertilizers, biofuel and source of natural products [10]. Additionally, marine macroalgae can be easily cultivated and have a great biotechnological potential. 

**Figure 1:**
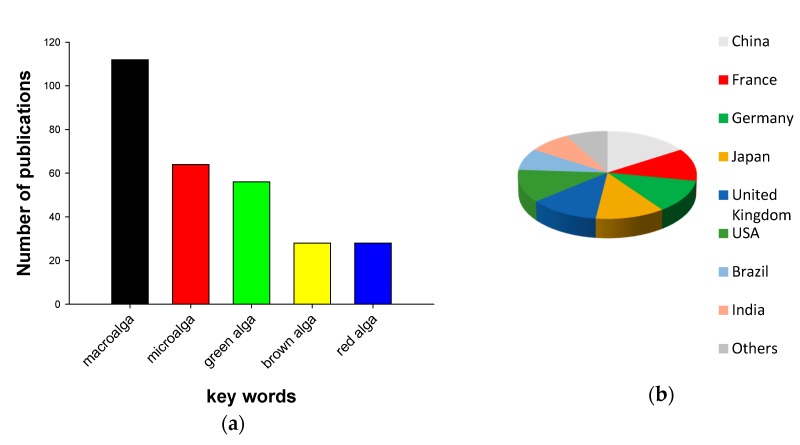
(**a**) Antifouling algal-related publications in the scientific literature. To access the frequency of publications, we ran a search on Scopus for the period from 2010 to February 2017. Our search terms include “antifouling”, plus other keywords; and (**b**) affiliations of researchers of antifouling algal-related publications. To access the frequency of publications, we ran a search on Scopus for the period from 2010 to February 2017. Our search terms include “antifouling alga” plus “natural product”.

Since 2010, more than 160 scientific publications about antifouling products from marine algae have been published (Figure 1a). These include articles, reviews and book chapters. An analysis of this literature suggested that most publications were about macroalgae, while researchers published articles dealing with microalgal antifouling compounds 1.7 times less frequently. Among macroalgal publications, the majority of articles were dealing with green algae (Figure 1a). Publications about brown and red algae were less abundant. Approximately 16% of all publications about antifouling (AF) compounds from algae were published by researchers from China (Figure 1b), and 76% of publications were produced by researchers from China, France, Germany, Japan, USA, and UK. 

Marine macroalgae are ubiquitous at rocky shores and have a high biotechnological potential. A comprehensive review about antifouling (AF) compounds from marine algae was published in 2004 [10]. After that, several reviews about compounds of marine organisms with potential AF properties have been published. These include several publications about antifouling natural products from all groups of marine organisms [8,11,12,13], as well as from marine microbes, including cyanobacteria and fungi [2,14,15,16]. The present publication is aimed to review scientific publications dealing with AF compounds of macroalgae from 2010 to February 2017, to examine current progress and highlight future trends. We review the antifouling compounds from green, brown, and red macroalgae, their mechanisms of action, and provide future perspectives.

## 2. Antifouling Compounds from Marine Macroalgae

### 2.1. Green Macroalgae (Chlorophyta)

**Figure 2:**
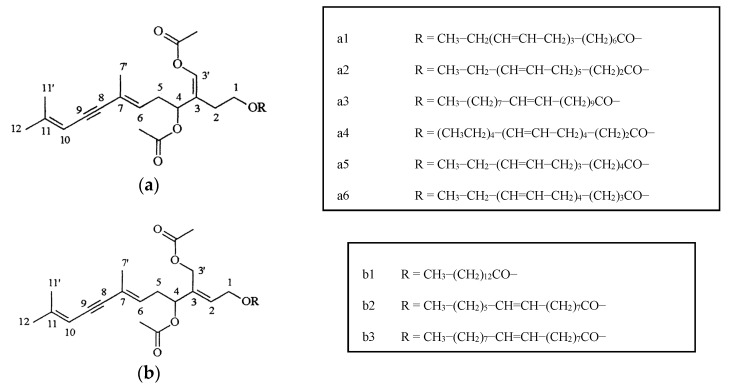
Acetylene sesquiterpenoid esters (**a**,**b**) from *Caulerpa prolifera*.

Chlorophyta algae belonging to the genera *Ulva, Codium, Caulerpa*, and *Ulva (Enteromorpha*), in particular, were studied for their antimicrobial (antibacterial and antialgal) effects in laboratory experiments (see Table 1). Only a few algal species were tested against bacterial quorum sensing (QS, see below). The compounds were rather undefined in these studies, ranging from alkaloid phenolic acids, to organic extracts and included, in one case, polar and non-polar extracts. Several structures of the identified substances were provided in Figure 2. Only the alga *Ulva rigida* was tested for, and showed, general AF activity [17]. In this study the investigators tested the polymeric replica of brown algae *Saccharina latissima* and *Fucus guiryi* doped with 3-bromo-5-(diphenulene)-2(5H)-furanone isolated from the green alga *U. rigida*. The study demonstrated that both chemical and physical properties of algae were important for antifouling defense [17]. Prabhakaran and co-workers [18] studied the antifouling potential of various extracts from seaweeds, seagrasses, and mangroves. When comparing the inhibitory activity of all extracts, mangrove plants had the highest inhibitory activity against tested biofilm-forming bacteria compared to seaweeds and seagrasses. In contrast, tests conducted with micro-fouling bacteria obtained from biofilms developed on PVC sheets showed that extracts of seaweeds had a better antimicrobial activity than those of seagrasses and mangroves [18]. Ethanol extracts of seaweed *U. reticulata* had maximal antibacterial activity against *Flavobacterium* sp. and a minimum activity against the remaining three biofilm bacteria of that study. The inhibitory activity was correlated with the major functional groups of the extracts, such as hydroxyl, amino, carbonyl and phosphoryl functionalities, aliphatic (fatty acids), NH_2_ (amide I and II). The authors claim that molecular bonds, such as O–H stretch, H-bond, C–H stretch, –C=C– stretch, C–O stretch, and C-Br stretch, were involved in the inhibitory activity of all the extracts. Bonds such as O–H stretch, H-bond, C–H stretch, –C=C– stretch, C–O stretch, and C–Br stretch were found in all the extracts [18]. Hence, compounds with such bonds can be considered as potential anti-biofilm molecules. 

### 2.2. Brown Macroalgae (Phaeophyta)

**Figure 3:**
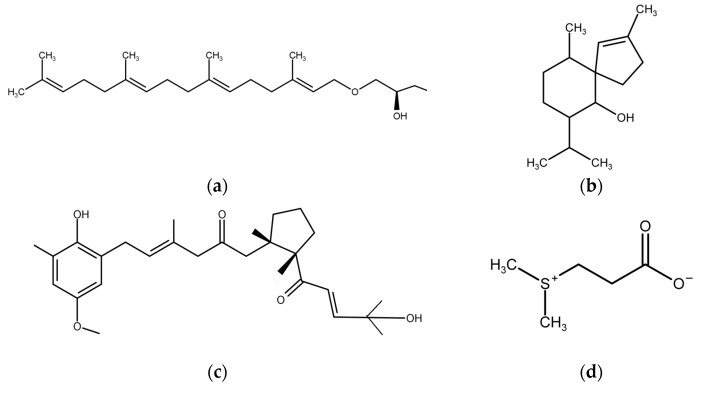

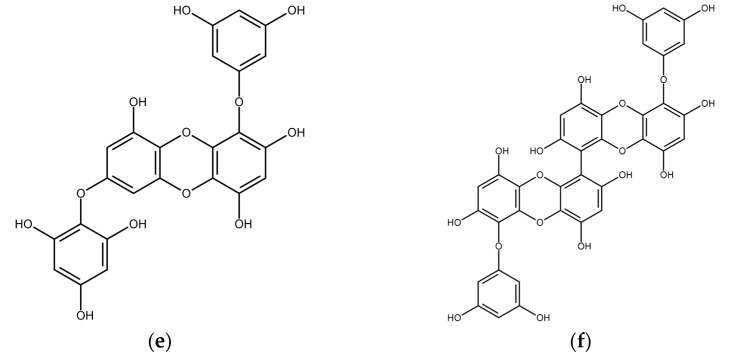
Antifouling compounds from brown macroalgae: (**a**) sn-3-*O*-(geranylgeranyl) glycerol from *Taonia atomaria* and *Dyctiota* sp.; (**b**) sesquiterpenoid (−)-gleenol from *T. atomaria*; (**c**) monocyclic meroditerpenoid from *Cystoseira tamariscifolia*; (**d**) dimethylsulphopropionate from *Fucus vesiculosus*; (**e**) 1-(3′,5′-dihydroxyphenoxy)-7-(2′′,4′′,6-trihydroxyphenoxy)-2,4,9-trihydroxydibenzo-1,4-dioxin; and (**f**) 6,6′-bieckol.

Brown algae were, by far, the best investigated macroalgae within the report frame of this review (see Table 2). They showed primarily anti-bacterial (including anti-QS) effects, followed by anti-algal, anti-diatom, and anti-larval effects. Most of the responsible compounds remained undefined, but were extracted primarily by alcoholic polar extracts (Table 2). Several of the identified substances causing AF are provided in Figure 3. Ethanol extract of the seaweed *Sargassum wightii* showed maximal antibacterial activity against *Flavobacterium* sp. and *Bacillus* sp., which was similar to that of extracts of the green algae *Ulva reticulata* and *Halimeda macroloba* [18]. Several investigators studied the seasonal variation of AF defense of *Fucus vesiculosus* [25,26,27]. It was found that the defense varied spatially and temporally. Surface extracts of the alga allowed the isolation of surface-attached AF compounds from *F. vesiculosus* that were identified as dimethylsulphopropionate (DMSP) and proline [28]. Several investigators studied AF compounds from *Sargassum* spp. (Table 2), which included phlorotannins [29], galactoglycerolipids [30], stigmasta-5,22-*E-*,28-triene-3β,24α-diol [31], and chromanols [32] (Figure 3). Extracts of the invasive *Sargassum muticum* were more effective against the growth of diatoms, bacteria, and the settlement of *Bugula neritina* larvae than native *Sargassum* species [33]. Similarly, in another study the anti-diatom effect of *S. muticum* extract was 10-fold lower than AF booster biocides, but algal extracts were less toxic [34].

### 2.3. Red Macroalgae (Rhodophyta)

In terms of AF activity, red algae provided the second best investigated macroalgal group (see Table 3). Several genera led by *Laurencia* spp. and *Asparagopsis* spp. were shown to have antimicrobial (particularly anti-bacterial, including anti-QS, and anti-diatom) effects, followed by spore, anti-larval and, generally, AF inhibition. It is interesting that fatty acid derivatives with AF activity, mainly docosane, hexadecanoic acid, and cholesterol trimethylsilyl ether, were not only produced and secreted by cortical cells, but also deposited on the surface of *Laurencia translucida* [44].

**Figure 4:**
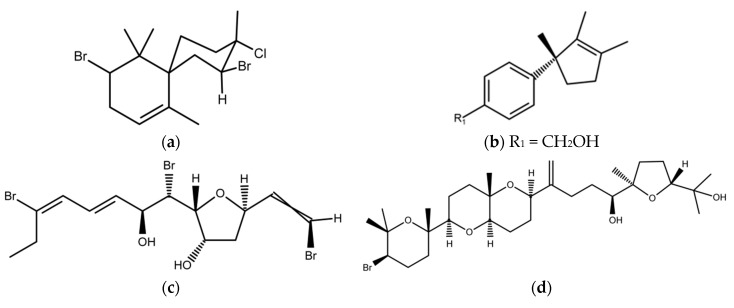

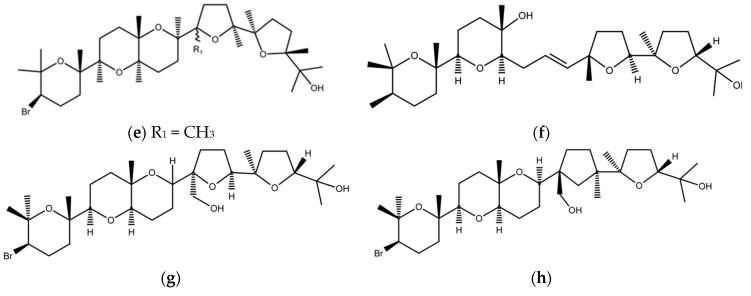
Some antifouling compounds from red macroalgae: (**a**) 2,10-dibromo-3-chloro-7-chamigrene from *Laurencia* obtusa; (**b**) 12-hydroxyisolaurene from *Laurencia obtusa*; (**c**) Omaezallene from *Laurencia* sp.; (**d**) Dehydrothyrsiferol; (**e**) Saiyacenols B; (**f**) Saiyacenols C; (**g**) 28-hydroxysaiyacenol B from *Laurencia viridis*; (**h**) 28-hydroxysaiyacenol A from *L. viridis*.

In most studies, only AF of extracts of red macroalgae was tested. The majority of the extracts with AF activity were polar (either methanol or ethanol). Several of the identified AF substances are provided in Figure 4. 2,10-dibromo-3-chloro-7-chamigrene 12-hydroxyisolaurene from *L. obtusa* inhibited barnacle *Balanus amphitrite* settlement at a concentration three-fold lower than the biocide copper sulfate [45]. *Laurencia* sp. also produced omaezallene, which, in the barnacle settlement assay, has an EC_50_ 0.22 μg/mL, while it shows a low toxicity LC_50_ of 4.8 µg/mL [46]. In another study, saiyacenols B and C, dehydrothyrsiferol, as well as 28-hydroxysaiyacenols B and A, were isolated from *L. viridis* [47]. AF activity of these compounds was investigated against bacteria, fungi, diatoms and algal spore settlement. All compounds at micromolar concentrations were effective only against diatoms *Navicula* cf. *salinicola* and *Cylindrotheca* sp., while 28-hydroxysaiyacenols B and A also inhibited the germination of *Gayralia oxysperma* spores.

## 3. Quorum Sensing Inhibitors from Macroalgae

QS is a population-density dependent communication between bacteria [53]. During this process, bacteria produce small signal QS molecules, which accumulate in the environment until, at a threshold concentration, the change of density-dependent behavior of bacteria is triggered [15]. Well-known examples of QS molecules are N-acyl homoserine lactones (AHLs) that are part of this intercellular signaling system in Gram-negative bacteria [54]. Bacterial quorum sensing (QS) has been proposed as a potential new approach for controlling biofouling [8]. Since the discovery of bacterial QS communication, many investigators have searched for molecules that can disrupt or block QS signaling in bacteria [55]. 

**Figure 5:**
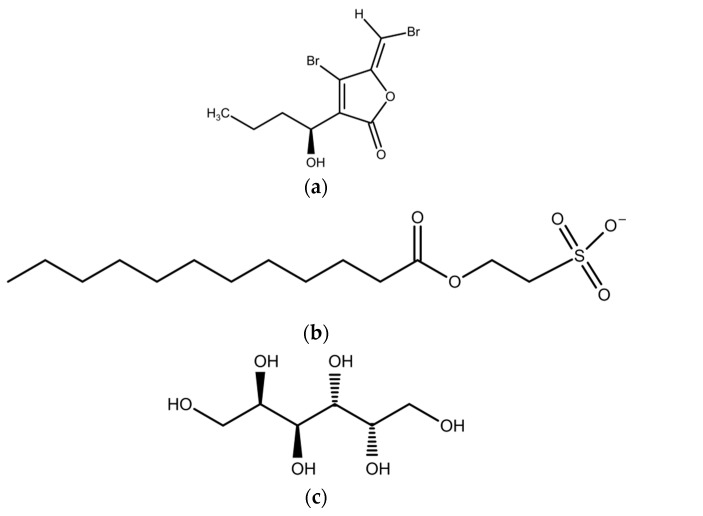
Quorum sensing (QS) inhibitors from marine macroalgae: (**a**) halogenated furanone from *Delisea pulchra*; (**b**) 2-dodecanoyloxyethanesulfonate from *Asparagopis taxiformis*; and (**c**) galactitol from *Spatoglossum* sp.

Marine macroalgae are able to stimulate, inhibit, or compromise QS signals in bacteria [56,57]. The first QS inhibitory compound was isolated from the marine red macroalga *Delisea pulchra* [57]. This alga secretes furanones that mimic bacterial AHL signals (Figure 5). Later studies have shown that other macroalgal species, as well, produce QS and biofilm formation inhibitors (see Table 1, Table 2 and Table 3). Jha et al. [50] studied 30 macroalgal species, but only 2-dodecanoyloxyethanesulfonate from the red alga *Asparagopis taxiformis* inhibited QS of the reporter strains *Chromobacterium violaceum* CV026 and *Serratia liquefaciens* MG44. In addition, compounds demonstrated significant toxicity, but QS inhibition was observed at non-toxic concentrations. Hypobromous acid produced by the brown alga *Laminaria digitata* interferes with bacterial QS signals and genes [58]. The brown alga *Spatoglossum* sp. produces the QS inhibitor dulcitol [59]. This compound compromised luminescence production of *E. coli*-based reporters in the presence of acyl homoserine lactones (QS signals). Batista and co-workers [21] found that about 91% of polar (methanol/water) extracts of tested macroalgae inhibited the QS of the reporter bacterium *C. violaceum* CV017. Additionally, polar extracts of algae were found to show considerable antibacterial activity exhibited against biofilm forming bacteria. The higher bioactivity of polar extracts could be due to a higher solubility of QS-inhibitory compounds in seawater that was used in this study. The minimal inhibitory concentrations (MICs) of non-polar extracts were 10- to 1000-fold higher than the effective concentrations, suggesting that the extracts were not toxic. Another study that used green, brown, and red macroalgae from the Brazilian coast showed that their extracts inhibited QS of the reporter *C. violaceum* CV017 [35]. Additionally, bacteria from the surface of the green algae *Ulva* sp. and *Colpomenia sinuosa* can inhibit QS and prevent biofouling [60]. There is no clear understanding about the true biosynthetic origin of QS inhibitors from macroalgae, which may be produced by the algae themselves, by their associated bacteria, or by both [56]. In most of cases, the mechanisms of QS inhibition by algal metabolites are not clear and need to be studied in the future. 

## 4. Potential of Macroalgal Extracts in Biological Synthesis of Nanoparticles

Nanotechnology is currently affecting many aspects of science and applied technologies, which include the design, synthesis, and manipulation of small structures for several applications, e.g., in medicine and life sciences. Nanoparticles and nanostructures are currently used in various applications, such as catalytic activity, water purification, chemical and biological sensors, and wireless electronic logic and memory schemes [61]. Particularly, metal and metal oxide nanoparticles, such as silver, gold, and platinum, have been used in the sector of bioelectronics, medicine, and pharmaceuticals [62]. Recently, antifouling activity of metal and metal oxide nanostructures has been reported [63,64,65,66]. 

Biological synthesis of nanoparticles is an emerging technical tool to address eco-friendly, cost effective, energy-efficient, and reliable production method of metal nanoparticles. Among these are silver nanoparticles (AgNPs), one of the most widely used due to their size, shape, and applications [67]. AgNPs are proven to have AF activity [64]. Chemical synthesis of metal nanoparticles requires reducing and capping agents, such as surfactants, which are toxic. In contrast, biological synthesis requires only extracts with a reducing agent, and a capping agent at low energy requirements [68]. Seaweeds can effectively be synthesized to metal nanoparticles [69]. Green synthesis of nanoparticles using seaweeds attracts significant research attention nowadays, which also holds for antifouling applications. Ramkumar et al. [70] synthesized biocompatible and functionalized silver nanoparticles by using an aqueous extract of the green seaweed *Ulva* (*Enteromorpha*) *compressa* as a reductant, as well as a stabilizing agent. They also demonstrated that these nanoparticles have strong antimicrobial and anticancer activity. Metal nanoparticles synthesized by macroalgae could potentially be utilized in AF applications. 

## 5. Antifouling Defense 

Macroalgae need several factors in order to survive in the marine environment. These include the availability of nutrients and light. They have to cope with grazing pressure, competition for space and resources, as well as parasites and diseases [71]. Biofouling on the surface of macroalgae (called epibiosis) leads to a reduction of algal access to light, gases and nutrients, and probably increases grazing and infections by pathogens [3]. Marine macroalgae evolved different chemical, physical and biological mechanisms to prevent epibiosis [8,11]. Understanding these ecological strategies is of importance for the successful development of AF technologies for marine installations. Macroalgae prevent biofouling by a combination of different, not only chemical, AF strategies. For example, experiments with polymeric surfaces mimicking thalli of *Saccharina latissima* and *Fucus guiryi* demonstrated that doping of such synthetic matrices with brominated furanones would increase their AF performance by 40% [17]. This clearly suggests that both chemistry and microtopography are important for the successful defense of algae from fouling. 

Marine macroalgae may have a complex, largely unknown, AF compound delivery system. This includes different structures at the thallus cortex, such as gland cells in *Delisea* and *Asparagopsis* species [72], intracellular organelles “corps en cerise” in some *Laurencia* species [73], and specific vacuoles, mevalonosomes, in *Plocamium brasiliense* [74].

Most of the research has been performed with common green (*Ulva* spp.), brown (*Fucus* spp., *Sargassum* spp., *Dyctiota* spp.), and red (*Ceramium* spp.) macroalgal species (Table 1, Table 2 and Table 3). As yet, macroalgae from tropical environments were poorly investigated. Species in tropical environments probably experience higher fouling pressure compared to temperate or polar species [75]. Thus, tropical and subtropical algal species may hide a high number of AF compounds [75,76]. Invasive macroalgal species can be another potent source of AF compounds. Recent studies suggested that extracts of the invasive alga *Sargassum muticum* from Japan have higher anti-bacterial, anti-diatom, anti-larval, and quorum sensing (QS) inhibitory activity in Oman waters compared to *Sargassum* spp., which are endemic here [33]. 

Most studies investigating AF activity of algal extracts or compounds in laboratory experiments were using monocultures of bacteria, diatoms, larvae of invertebrates, and spores of algae. To the contrary, there is a multitude more of different micro- and macro-fouling organisms in the marine environment [77]. It is estimated that less than 2% of bacteria taken from environmental samples can be grown in laboratory conditions [78]. Thus, successful performance of AF compounds in the laboratory does not guarantee that these compounds will be active under field conditions. In the laboratory, extracts of *Sargassum* spp. inhibited the growth of pathogens and environmental bacteria, while in field experiments these extracts embedded in a Phytagel matrix stimulated the growth of marine bacteria [33]. 

Investigators commonly use the whole thalli of macroalgae in order to extract bioactive compounds under laboratory conditions. Such methods do not allow for understanding the production of bioactive compounds and their concentrations under in vivo conditions. Moreover, in most cases it is not possible to compare the effective concentrations of algal AF compounds with ones that are produced in the environment. Only a few studies developed and used gentle soaking techniques to extract algal surface metabolites without destroying the integrity of algal cells, which leads to contamination with intracellular compounds [25,28,36,37]. Gentle soaking techniques include quick (5–10 s) soaking of algal thalli in organic solvents, like hexanes or dichloromethane [26,79]. Recent studies demonstrated new AF mechanisms that included the production of fatty acid derivatives by corticoid cells of the red alga *Laurencia translucida* and their deposition on the surface of the algal thallus [44]. A novel, robust method of the extraction of surface-bound metabolites was proposed [80]. It is based on the powdering of wet algal surfaces with C18 solid phase material. Later, the authors recovered algal metabolites and analyzed them through liquid or gas chromatography coupled with mass spectrometry. The development of new methods is required to study the production of AF compounds by algae in vivo and estimate their realistic effective concentrations.

## 6. Role of Epibiotic Organisms

**Figure 6:**
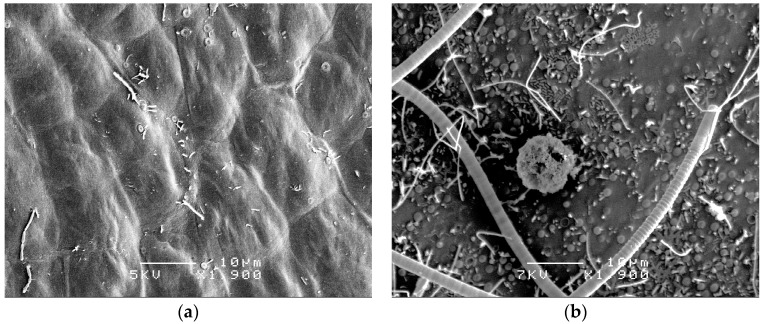
Biofouling on the surface of the green alga *Ulva reticulata*: (**a**) scanning electron microscopy (SEM) photo of the healthy part of the alga with low bacterial fouling; and (**b**) SEM photo of the old part of the same alga which was heavily fouled with fungi, microalgae, and bacteria. Magnification = 1900×; scale bar = 10 µm.

Surfaces of marine macroalgae are commonly covered by different species of bacteria, microalgae, and fungi (epibionts), whose composition and density vary with environmental conditions and algal parts ([25]; Figure 6). In some cases, several epibionts are known to penetrate the thalli of macroalgae [76]. There is growing evidence that microbial communities associated with algae are different from other substrata and surrounding waters (see review [3]). Several studies demonstrated that epibionts associated with algae can produce antifouling compounds that defend their hosts [16,81,82,83]. For example, *Vibrio* sp. isolated from the green alga *Ulva reticulata* produces an AF waterborne compound [84,85]. Another study demonstrated that most of the bacteria isolated from the surface of the brown alga *Colpomenia sinuosa* produce QS inhibitory compounds [60].

Investigators usually do not take into account that epibiotic microorganisms are associated with macroalgae (Figure 6). This may result in the extraction of both algal and microbial metabolites. In contrast to previous studies that used extracts of algae and their natural microflora [50,55], Batista et al. [21] used an ethanol treatment [86] to eliminate surface-attached bacteria and diatoms. Their data indicated that microorganisms living on the surface of some algae could be responsible for the production of QS inhibitory compounds [21]. Although extracts of *U. fasciata*, *Caulerpa racemosa*, and *Codium* sp. had some QS inhibitory activity in the absence of microbes, the activities of extracts from these algae with microbes were significantly higher [21]. Polar extracts of those macroalgae mentioned above with attached microorganisms, but never without them, inhibited the attachment of *Pseudomonas aeruginosa* PAO1. This confirms the notion that epibiotic bacteria are important for the production of antibacterial compounds [87]. Other studies also have shown the importance of bacteria isolated from the surface of seaweeds for protecting the host from fouling by other organisms [3,56]. On the other hand, microorganisms located in the thalli of marine algae probably would not be responsible for the production of surface-associated or excreted AF compounds [76].

## 7. Conclusions and Future Outlook

This review demonstrated that marine macroalgae provide a potent source of novel AF compounds. Additionally, macroalgae can be used for green synthesis of nanoparticles that can be utilized in AF applications. In most cases, researchers tested crude extracts of algae, but neither isolated nor elucidated the structure of AF compounds (see Table 1, Table 2 and Table 3). Thus, it is difficult to tell if these extracts contain novel AF compounds or not. In contrast to a Scopus publication search (Figure 1), we found that most of AF compounds were isolated from brown and red algae, but not from green macroalgae. This could be explained by the fact that, traditionally, these groups were studied more intensively or reflect the fact that these algae could be better defended against micro- and macrofouling. In previous studies many authors have failed to elucidate AF chemicals compounds from green algae from different regions [88,89]. Moreover, previous screening programs indicated that red, but not brown, seaweeds are the most potent in terms of production of AF compounds [88,89]. Isolation of bioactive compounds from algae involves a bioassay-guided approach, whereas imaging-based high-content screening (HCS) has been proposed as a promising tool for screening of algal bioactive potential [90]. However, screening and isolation of more AF compounds from macroalgae are needed in the future.

Isolation of these potentially-important biogenic compounds from marine algae is currently very expensive and time consuming. Combinatorial genetic or metabolic engineering [91], or hybrids [92], might be possible remedies for this problem. In addition to offering a secure supply of naturally-occurring metabolites, such technologies could be used to produce more-diverse chemicals. Although research is relatively new within this area, with only a few studies published to date, it seems that soon it will be possible to transfer the genes responsible for the production of these active secondary metabolites from one organism to more productive organisms. Then, sustainable compound production will become cheaper, fostering sustainable antifouling practices in aquaculture [92] or in the food industry [93].

Progress in isolating and producing marine algal bioactive compounds is expected to involve the integration of biochemistry-validated post-genomic methods and techniques, as well as smart bioprocessing. Levels of toxicity and capacities for biological degradation of these compounds in the aquatic environment need to be studied before they will be applicable in AF coatings for the prevention of biofouling. Once biogenic compounds become incorporated into AF paints, monitoring needs to be done over longer periods. The identification of these biogenic compounds with antifouling properties requires a wide range of expertise from the fields of biology, as well as chemistry. Metabolic engineering may provide a possible approach for future exploitation of secondary metabolites with AF properties from marine macroalgae.

There are several issues to be studied with priority concerning AF compounds from macroalgae. In particular, new antifouling compounds from tropical macroalgae should be isolated and tested in field experiments. Compounds need to be isolated in an ecologically-relevant way in order to prevent contamination by algal intracellular metabolites and compounds from epibiotic microorganisms. Finally, a multidisciplinary approach involving organic chemistry, biology, microbiology, and ecology specialists is required in the search for promising AF compounds and their biotechnological applications.

## Figures and Tables

**Table 1:** Antifouling compounds from green macroalgae (Chlorophyta).

Algae	Type of Activity	Compound	Reference ^1^
*Ulva rigida*	Antifouling	3-bromo-5-(diphenylene)-2(5H)-furanone	[17]
*Ulva pertusa*	Anti-algal	Alkaloid phenolic acid	[19]
*Ulva reticulate Halimeda macroloba*	Anti-bacterial	Organic extract ^2^	[18]
*Ulva lactuca*	Anti-bacterial	Organic extract ^2^	
*Codium fragile*	Anti-algal	Organic extract ^2^	[20]
*Caulerpa racemosa Codium* spp. *Ulva (Enteromorpha) fasciata*	Anti-QS, Anti-bacterial	Polar and non-polar extracts ^2^	[21]
*Ulva* sp.	Antifouling	β-carotene	[22]
*Caulerpa prolifera*	Antibacterial, antialgal	Acetylene sesquiterpenoid esters	[23]
*Chlorococcum* *humicola*	Antibacterial and anti *Aspergillus*	Chlorophyll a and b	[24]

^1^ The data are from research published from January 2010 to February 2017. ^2^ The structure is unknown. QS: quorum sensing.

**Table 2:** Antifouling compounds from brown macroalgae (Phaeophyta).

Algae	Type of Activity	Compound	Reference ^1^
Native and invasive *Sargassum* spp.	Anti-QS Anti-larval Anti-diatom	Non-polar extracts ^2^	[33]
*Sargassum* spp.	Anti-algal	Phlorotannin	[29]
*Sargassum muticum*	Anti-diatom	Ethanol and Dichlormethane extracts ^2^	[34]
*S. muticum*	Anti-bacterial	Galactoglycerolipids	[30]
*S. thunbergii*	Anti-larval	Stigmasta-5,22-*E-*,28-triene-3β,24α-diol	[31]
*S. horneri*	Anti-bacterial Anti-larval Anti-diatom	Chromanols	[32]
*S. vulgare Padina* sp.	Anti-QS Anti-bacterial	Polar and non-polar extracts ^2^	[35]
*S. furcatum Dyctiota* sp.	Anti-QS Anti-bacterial	Polar and non-polar extracts ^2^	[21]
*Taonia atomaria*	Anti-bacterial	Sesquiterpenoids	[36]
*Taonia atomaria*	Anti-bacterial Anti-larval	Sesquiterpenoid (−)-gleenol sn-3-*O*-(geranylgeranyl)glycerol	[37]
*Padina tetrastromatica*	Anti-bacterial Anti-diatom Anti-mussel	Methanol extracts (fatty acids) ^2^	[38]
*Cystoseira tamariscifolia*	Anti-bacterial Anti-fungal, Anti-larval, Anti-algal	Cystophloroketals A-E monocyclic meroditerpenoid	[39]
*Halidrys siliquosa*	Anti-bacterial	Methanolic extracts ^2^	[40]
*Dyctiota* spp.	Anti-bacterial	Diterpenes 1-*Ο*-octadecenoylglycerol sn-3-*Ο*-(geranylgeranyl)glycerol	[41]
*Dyctiota* sp.	Anti-bacterial Anti-algal	Extract ^2^	[42]
*Bifurcaria bifurcata*	Anti-bacterial Anti-fouling	Acyclic linear diterpenoids	[43]
*Fucus vesiculosus*	Anti-bacterial	Surface extracts ^2^	[25]
*F.vesiculosus*	Anti-bacterial	Surface extracts ^2^	[26]
*F.vesiculosus*	Anti-bacterial	Dimethylsulphopropionate Proline	[28]
*Laurencia johnstonii*	Anti-bacterial	Organic extract ^2^	[20]

^1^ The data are from research published from January 2010 to February 2017. ^2^ The structure is unknown. QS: quorum sensing.

**Table 3:** Antifouling compounds from red macroalgae (Rhodophyta).

Algae	Type of Activity	Compound	Reference ^1^
Crustose coralline algae	Anti-algal	Methanol extract ^2^	[48]
*Galdieria sulphuraria*	Antifouling	Floridoside	[49]
*Laurencia translucida*	Anti-bacterial	Fatty acid derivates	[44]
*Laurencia obtusa*	Anti-larval	2,10-dibromo-3-chloro-7-chamigrene 12-hydroxyisolaurene	[45]
*Asparagopsis taxiformis*	Anti-bacterial Anti-QS	Methanol extract 2-dodecanoyloxyethanesulfonate	[50]
*A. taxiformis*	Anti-bacterial Anti-algal	Extract ^2^	[51]
*Ceramium botryocarpum*	Anti-diatom	Ethanol and Dichlormethane extracts ^2^	[34]
*Chondrus crispus*	Anti-algal Anti-bacterial	Crude extracts ^2^	[52]
*Pterocladiella capillacea* *Spyridia aculeata*	Anti-QS Anti-bacterial	Polar and non-polar extracts ^2^	[35]
*P. capillacea Peyssonnelia capensis Spyridia* spp.	Anti-QS Anti-bacterial	Polar and non-polar extracts ^2^	[21]
*Laurencia* sp.	Antifouling	Omaezallene	[46]
*Laurencia translucida*	Antifouling	Fatty acid	[44]
*Laurencia viridis*	Anti-diatom	Dehydrothyrsiferol	[47]
*L. viridis*	Anti-diatom	Saiyacenols B	[47]
*L. viridis*	Anti-diatom	Saiyacenols C	[47]
*L. viridis*	Anti-algal	28-hydroxysaiyacenols B and A	[47]

^1^ The data are from research published from January 2010 to February 2017. ^2^ The structure is unknown. QS: quorum sensing.
